# Investigation of treatment satisfaction and health-related quality of life after add-on to metformin-based therapy in patients with type 2 diabetes

**DOI:** 10.3389/fpubh.2023.1152284

**Published:** 2023-04-11

**Authors:** Yu-Wen Chang, Feng-Chin Shen, Chung-Yu Chen

**Affiliations:** ^1^School of Pharmacy, Kaohsiung Medical University, Kaohsiung, Taiwan; ^2^Department of Pharmacy, Kaohsiung Chang Gung Memorial Hospital, Kaohsiung, Taiwan; ^3^Division of Endocrinology and Metabolism, Department of Internal Medicine, Kaohsiung Chang Gung Memorial Hospital, Kaohsiung, Taiwan; ^4^College of Medicine, Chang Gung University, Taoyuan, Taiwan; ^5^Department of Pharmacy, Kaohsiung Medical University Hospital, Kaohsiung, Taiwan; ^6^Department of Medical Research, Kaohsiung Medical University Hospital, Kaohsiung, Taiwan

**Keywords:** type 2 diabetes, ADDQoL, C-SOADAS, quality of life, treatment satisfaction

## Abstract

**Background:**

The complexity of oral antidiabetic drug (OAD) regimens affects the quality of life (QOL) and treatment satisfaction. However, data on the QOL of patients with type 2 diabetes mellitus (T2DM) receiving metformin-based OAD treatment in Asia are limited. Therefore, this study aimed to evaluate the QOL and treatment satisfaction and explore the influencing factors and their correlations among patients with T2DM receiving metformin-based OADs.

**Methods:**

This was a cross-sectional study conducted at the Outpatient Department of Metabolism and Endocrinology at a medical center in Taiwan. Data were collected using the Audit of Diabetes-Dependent Quality of Life (ADDQoL) and the Chinese version of the Satisfaction with Oral Anti-Diabetic Agent Scale (C-SOADAS) questionnaires from patients with T2DM using metformin. The outcomes were analyzed by group and stratified based on the use of two, three, and more than three OADs. The level of agreement between the questionnaires was analyzed using Spearman’s rank correlation coefficient.

**Results:**

A total of 153 patients with T2DM using metformin were included in this study. The average weighted impact score in the ADDQoL was −2.11, with no significant differences between the three groups. The C-SOADAS score showed a significant difference between the groups using two, three, and more than three OADs (21.42 [1.98] vs. 20.43 [2.09] vs. 19.00 [2.24], *p* < 0.0001). The ADDQoL and C-SOADAS scores showed low correlations between patients’ QOL and treatment satisfaction. However, the impact of diabetes on specific aspects of life was negatively correlated with the total C-SOADAS scores.

**Conclusion:**

In Taiwan, a significantly greater effect on QOL was observed among patients with fewer OAD classes and higher treatment satisfaction. This study provides local evidence from self-reporting outcomes of patients with T2DM. Further studies focusing on different populations and treatment regimens for QOL are needed.

## Introduction

Type 2 diabetes (T2D) has been one of the fastest growing diseases in the past decade, and its complications have a major impact on health-related quality of life (HRQoL) (1, 2). Previous studies showed that reducing the development of hypoglycemia can improve patients’ treatment satisfaction and HRQoL and attain glycemic control (3–6). Furthermore, increasing treatment satisfaction and HRQoL in patients with T2D are associated with a lower occurrence of diabetes complications and mortality (7, 8).

Metformin is usually the first-line therapy for patients with T2D. Adding second-line regimens, including various combinations of oral antidiabetic drugs (OADs), when glycated hemoglobin (HbA1c) does not reach the target is recommended by current guidelines. However, medical benefits and risks should be considered when choosing second-line OADs, especially given the current limited information on treatment satisfaction and HRQoL. Previous studies showed that improving patients’ treatment satisfaction can enhance treatment efficacy and adherence, and optimal glycemic control can reduce comorbidities and improve HRQoL. However, the high regimen complexity of OADs may lower treatment satisfaction and HRQoL due to the risk of side effects (9).

According to pharmacoepidemiology research, 87.5% of outpatients with diabetes use OADs in Taiwan (10). However, most research on OAD strategies in Taiwan has focused on diabetes control and treatment-related adverse events. Furthermore, data on patient-reported outcomes regarding treatment satisfaction and HRQoL after the addition of the second-line OAD strategy are limited. Thus, this study aimed to survey the treatment satisfaction and HRQoL for patients with T2D receiving add-on therapy to a metformin-based regimen.

## Methods

### Study population

Patients with T2D who visited endocrinology and metabolism outpatient clinics at Kaohsiung Chang Gung Memorial Hospital, a medical center in southern Taiwan, between April 2020 and June 2021 were enrolled. The inclusion criteria included patients who had been using metformin-based therapy for at least 12 weeks and who were receiving add-on therapy during the outpatient visit (index date). The exclusion criteria included patients who received injection therapy, including glucagon-like peptide-1 receptor agonist or insulin, had received chemotherapy within 6 months before the index date, had no willingness to fill out the consent form or questionnaire, and had a diagnosis of type 1 diabetes, gestational diabetes, or cognitive impairment. The included patients were asked to complete the Chinese version of the Audit of Diabetes-Dependent Quality of Life (ADDQoL) (11, 12) for Taiwan and the Chinese version of the Satisfaction with Oral Anti-Diabetic Agent Scale (C-SOADAS) (13) at the index date and 3 months after the index date, respectively.

This study was approved by the Institutional Review Board of the Chang Gung Medical Foundation (no. 202000024B0C501). All participants provided written informed consent.

### Sample size calculation

Daniel’s (14) sample size formula was used to calculate the minimum sample size, where 92 patients with T2D were considered a representative sample size for this study. Based on the previously published data on T2D prevalence and more than one OAD in Taiwan, a prevalence (P) of 60%, a desired precision of 10%, and a 95% confidence level were used (10).

### Questionnaire measurement

In this study, the ADDQoL and C-SOADAS questionnaires in Taiwan were used to measure the diabetes-specific quality of life (QOL) and treatment satisfaction, respectively. The translated versions of these questionnaires were analyzed for reliability and validity, and all authors agreed to use them as research instruments for this study (11–13).

The Taiwanese version of the ADDQoL is a widely used tool for assessing diabetes-related QOL and consists of two overview items to measure generic QOL and 19 specific domains of life. The product of the impact rating and importance rating score for each domain is the weighted impact score, and the weighted impact scores are added and divided by the number of applicable domains to yield the overall average weighted impact (AWI) score, which ranges from −9 (maximum negative impact) to +3 (maximum positive impact). Negative AWI scores indicate that diabetes has a significant negative impact on QOL.

The Taiwan version of the C-SOADAS is a tool for evaluating treatment satisfaction based on a five-item scale, focusing on concepts related to satisfaction with OADs among patients with type 2 diabetes mellitus (T2DM), including (1) ability to control blood sugar, (2) effect on weight, (3) tolerability of the side effects, (4) convenience of drug taking, and (5) overall satisfaction. Each item was scored on a 5-point scale (ranging from 1 to 5). After adding up the scores of the five items, with 25 being the highest score, higher scores indicate higher satisfaction with OADs.

All participants were referred to the study by physicians. The study aim, methods, and consent form contents were explained clearly to eligible subjects by the researchers before inclusion. The questionnaire was completed after the consent form was completed and signed. The patients were allowed to complete the questionnaire by themselves during the index date. The questionnaires were administered once to each participant.

### Outcomes

The primary outcome was the ADDQoL and C-SOADAS questionnaire scores, and the secondary outcome was ascertained to assess the convergent validity of the questionnaire correlations for ADDQoL and C-SOADAS.

### Comorbidities and covariables

Basic data, including demographics, comorbidities, and laboratory values, were collected within 1 year from the index date and were based on the most recent data available. Demographic data included age, sex, duration of diabetes, family history of diabetes, smoking status, alcohol status, self-monitoring of blood glucose levels, and body mass index. Comorbidities included hypertension, dyslipidemia, cerebra/cardiovascular disease, peripheral vascular disease, neurological disease, retinopathy, and nephropathy. Laboratory data included HbA1c (<7% and ≥ 7%) and urine albuminuria-to-creatinine ratio (UACR) (<30, 30–300, and > 300). The use of fixed-dose combination OADs was evaluated based on the index date prescription.

### Statistical analysis

Basic data and questionnaire scores were presented as mean (standard deviation) for normally distributed variables and median (interquartile range) for non-normally distributed continuous variables. Categorical variables were presented as numbers and percentages and analyzed using the Chi-square test. Study participants were divided into three groups according to the number of OADs they used (two, three, and more than three OADs). The difference in C-SOADAS and ADDQoL items was determined using one-way ANOVA. The levels of agreement between questionnaires were analyzed using Spearman’s rank correlation coefficient, which was interpreted as follows: <0.300, low correlation; 0.301–0.700, moderate correlation; and >0.701, high correlation. Data processing was performed using Microsoft Excel, and statistical analysis was performed using SPSS software.

## Results

**Figure 1:**
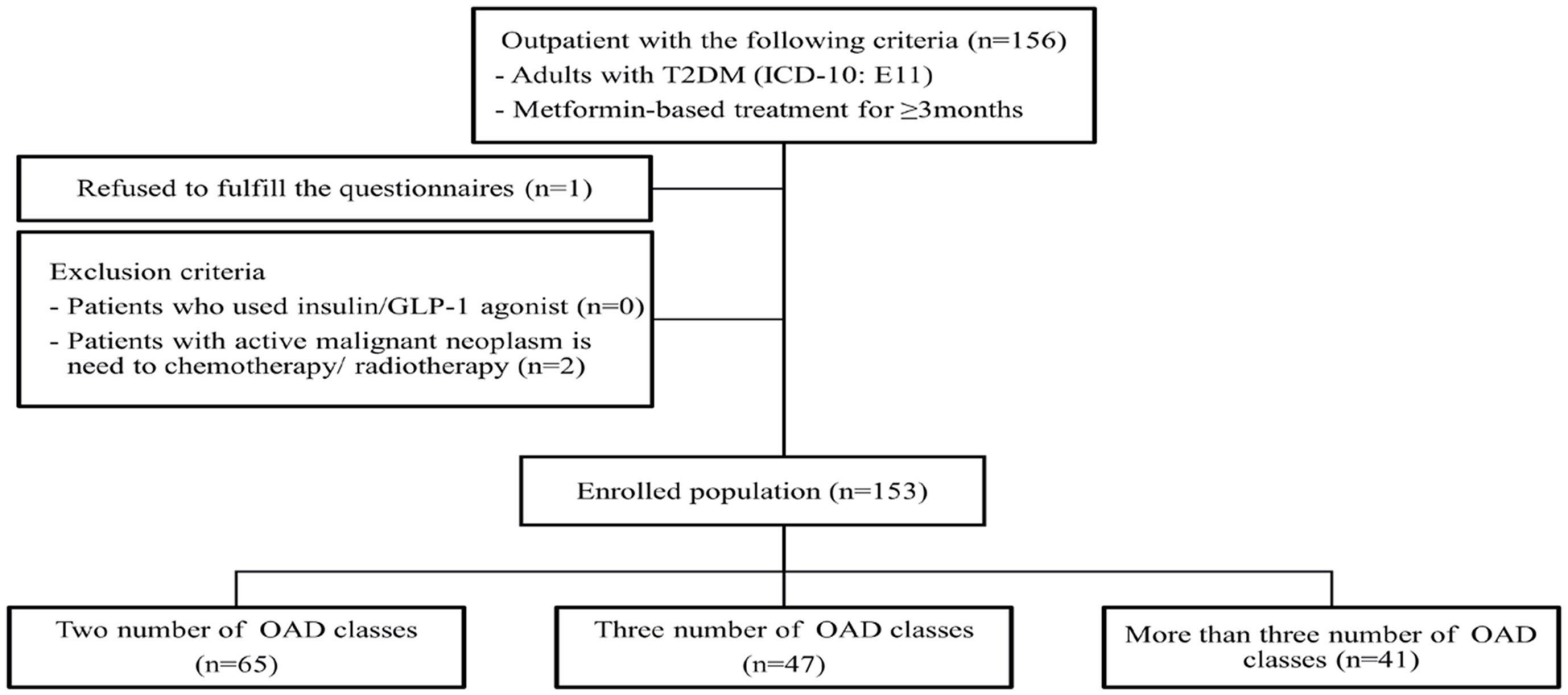
Study flow diagrams. T2DM, type 2 diabetes mellitus; OADs, oral anti-diabetic drugs; ICD-10, international classification of disease 10th revision.

A total of 156 patients with T2D were enrolled. Of the 156 patients, three were excluded (one refused to complete the questionnaire, and two were diagnosed with malignancy). Thus, 153 participants were included in the analysis (Figure 1). The characteristics of the total and subgroups of the study population are shown in Table 1. The mean age was 60.4 years, and most of the patients were 55–69 years old. The mean duration of T2D was 9.2 years, and 64.7% of the patients had a family history of diabetes. Only 43.79% of the patients had the habit of self-monitoring their blood glucose levels. Most of the patients had no history of smoking (81.70%) or alcohol consumption (81.05%), and nearly half of them (47.75%) were obese. Hypertension (51.63%) and dyslipidemia (89.54%) were the most prevalent comorbidities. In this study, the proportion of fixed-dose combination OADs was 71.24%.

**Table 1:** Characteristics of all patients and stratified by the different oral antidiabetic drug groups.

Variable	Total (*n* = 153)	2 OADs (*n* = 65)	3 OADs (*n* = 47)	>3 OADs (*n* = 41)	*p* value
DM duration	9.22 (5.70)	7.32 (4.86)	10.17 (6.43)	11.07 (5.29)	0.002
Age(years)	60.39 (10.14)	61.32 (9.46)	58.72 (11.41)	60.84 (9.52)	0.388
Gender					0.035
Female	65 (42.48)	35 (53.85)	18 (38.30)	12 (29.27)	
Male	88 (57.52)	30 (46.15)	29 (67.70)	29 (70.73)	
DM family history					0.870
No	42 (27.45)	18 (27.69)	14 (29.79)	10 (24.39)	
Yes	99 (64.71)	43 (66.15)	28 (59.57)	28 (68.29)	
Unknown	12 (7.84)	4 (6.15)	5 (10.64)	3 (7.32)	
Smoke					0.036
Current	15 (9.80)	2 (3.08)	4 (8.51)	9 (21.95)	
Past	13 (8.50)	5 (7.69)	5 (10.64)	3 (7.32)	
Never	125 (81.70)	58 (89.23)	38 (80.85)	29 (70.73)	
Alcohol					0.132
Yes	29 (18.95)	9 (13.85)	8 (17.02)	12 (29.27)	
Never	124 (81.05)	56 (86.15)	39 (82.98)	29 (70.73)	
Self-monitoring blood glucose	67 (43.79)	32 (49.23)	17 (36.17)	18 (43.90)	0.389
Body mass index					0.021
Underweight	2 (1.31)	2 (3.07)	-	-	
Normal	34 (22.22)	18 (27.69)	14 (29.79)	2 (4.88)	
Overweight	47 (30.72)	21 (32.81)	10 (21.28)	16 (39.02)	
Obese	70 (45.75)	24 (36.92)	23 (48.94)	23 (56.10)	
Fixed-dose combination OADs					<0.0001
Yes	109 (71.24)	25 (38.46)	45 (95.74)	39 (95.12)	
No	44 (28.76)	40 (61.54)	2 (4.26)	2 (4.88)	
DM related disease					
Hypertension	79 (51.63)	27 (41.54)	24 (51.06)	28 (68.29)	0.027
Dyslipidemia	137 (89.54)	57 (87.69)	41 (87.23)	39 (95.12)	0.393
Cerebra/cardiovascular disease	10 (6.54)	4 (6.15)	2 (4.26)	4 (9.76)	0.649
Peripheral vascular disease	2 (1.31)	1 (1.54)	0 (0.00)	1 (2.44)	0.737
Neuropathy	14 (9.15)	3 (4.62)	6 (12.77)	5 (12.20)	0.218
Retinopathy	2 (1.31)	0 (0.00)	2 (4.26)	0 (0.00)	0.164
Nephropathy	60 (39.22)	20 (30.77)	19 (40.43)	21 (51.22)	0.108
Plasma glucose (mg/dL)	163.16 (50.62)	124.31 (48.06)	159.62 (46.68)	181.27 (55.31)	0.023
HbA1c (%)	7.35 (0.99)	7.11 (0.95)	7.26 (0.99)	7.79 (0.93)	0.004
<7%	54 (35.29)	26 (40.00)	21 (44.68)	7 (17.07)	0.012
≥7%	99 (64.71)	39 (60.00)	26 (55.32)	34 (82.93)	
UACR (mg/g)					0.093
<30	109 (71.71)	52 (81.25)	34 (72.34)	23 (56.10)	
30–300	31 (20.39)	9 (14.06)	9 (19.15)	13 (31.71)	
>300	12 (7.89)	3 (4.69)	4 (8.51)	5 (12.20)	

Values are displayed as *n* (%), mean ± standard deviation. DM, diabetes mellitus; HbA1c, glycated hemoglobin A1c; OADs, oral antidiabetic drugs.

Based on the participant classification (Table 1), the mean duration of T2DM was 7.3 (4.9) years for the group with two OADs, 10.2 (6.4) years for the group with three OADs, and 11.1 (5.3) years for the group with more than three OADs, with a significant difference among the three groups (*p* = 0.0015). The proportion of patients with obesity was the highest in the group with more than three OADs (56.1%), with a significant difference between the three groups (*p* = 0.0214). The group with more than 3 OADs had the highest proportion of hypertension (68.3%). Family history of diabetes mellitus (DM), alcohol consumption, and home self-monitoring of blood glucose did not differ significantly among the three groups. In glycemic control, a significant difference in HbA1c levels was observed between the three groups (7.19% vs. 7.19% vs. 7.79%, *p* = 0.0035).

The ADDQoL scores are shown in Tables 2, 3. The mean and overall QOL scores were 0.86 (0.84) and 0.88 (0.86) for the group with two OADs, 0.79 (0.75) for the group with three OADs, and 0.92 (0.91) for the group with more than 3 OADs. No significant difference in the overall QOL was observed among the three groups (*p* = 0.7274). The mean scores for the impact of diabetes on specific life aspects were −1.68 (0.72) in all patients and −1.54 (0.73) for the group with two OADs, −1.87 (0.71) for the group with three OADs, and −1.68 (0.69) for the group with more than three OADs. The AWI scores were −2.11 (1.08) in the total participant population and −2.05 (1.08), −2.10 (0.90), and −2.23 (1.27) in the three groups, respectively, with no significant differences between the three groups. The group with more than three OADs had a higher negative impact on close personal relationships (−3.53) than the other two groups (*p* = 0.0433).

**Table 2:** ADDQoL and C-SOADAS of all participants.

Item	All participants (*n* = 153)
**ADDQoL**
Overview questions	
Present QoL	0.86 ± 0.84
Diabetes-dependent QoL score	−1.68 ± 0.72
19 domain-specific items	
Leisure activities	−1.78 ± 1.71
Working life	−1.69 ± 1.66
Journeys	−1.75 ± 1.77
Holidays	−1.98 ± 1.81
Physical health	−2.38 ± 1.86
Family life	−3.46 ± 2.34
Friendship and social life	−1.35 ± 1.80
Close personal relationship	−2.78 ± 2.12
Sex life	−1.85 ± 1.89
Physical appearance	−1.90 ± 1.88
Self-confidence	−1.51 ± 1.92
Motivation	−1.48 ± 1.55
People’s reaction	−0.76 ± 1.17
Feeling about future	−3.49 ± 2.33
Financial situation	−0.75 ± 1.36
Living conditions	−0.59 ± 1.16
Dependence on others	−1.48 ± 1.67
Freedom to eat	−4.75 ± 2.75
Freedom to drink	−4.57 ± 3.00
Average weighted impact score	−2.11 ± 1.08
**C-SOADAS**
Q1: Ability to control blood sugar	4.20 ± 0.76
Q2: Effect of weight	3.90 ± 0.63
Q3: Tolerability of the side effect	4.29 ± 0.53
Q4: Convenience of drug taking	3.86 ± 0.76
Q5: Overall satisfaction	4.23 ± 0.58
Total C-SOADAS score	20.46 ± 2.29

Value is displayed as mean ± standard deviation. ADDQoL, Audit of Diabetes-Dependent Quality of Life; C-SOADAS, Chinese version of the Satisfaction with Oral Antidiabetic drugs; QoL, quality of life; AWI, Average weighted impact score.

**Table 3:** ADDQoL score in different oral antidiabetic drug groups.

Item	2 OADs (*n* = 65)	3 OADs (*n* = 47)	>3 OADs (*n* = 41)	*p* value
**Overview questions**
Present QoL	0.88 ± 0.86	0.79 ± 0.75	0.92 ± 0.91	0.727
Diabetes-dependent QoL score	−1.54 ± 0.73	−1.87 ± 0.71	−1.68 ± 0.69	0.053
**19 domain-specific items**
Leisure activities	−1.54 ± 1.43	−1.81 ± 1.73	−2.15 ± 2.05	0.203
Working life	−1.52 ± 1.42	−1.68 ± 1.39	−1.96 ± 2.25	0.623
Journeys	−1.60 ± 1.52	−1.89 ± 1.88	−1.80 ± 2.03	0.669
Holidays	−1.66 ± 1.58	−2.07 ± 1.64	−2.44 ± 2.30	0.1351
Physical health	−2.38 ± 1.81	−2.45 ± 1.73	−2.29 ± 2.11	0.928
Family life	−3.14 ± 2.17	−3.96 ± 2.56	−3.38 ± 2.30	0.192
Friendship and social life	−1.31 ± 1.76	−1.38 ± 1.76	−1.37 ± 1.96	0.974
Close personal relationship	−2.39 ± 1.98	−2.66 ± 1.55	−3.53 ± 2.64	0.043^*^
Sex life	−1.50 ± 1.72	−1.69 ± 1.42	−2.61 ± 2.38	0.042^*^
Physical appearance	−1.91 ± 1.95	−1.80 ± 1.67	−2.00 ± 2.04	0.890
Self-confidence	−1.75 ± 2.13	−1.21 ± 1.53	−1.46 ± 1.98	0.337
Motivation	−1.74 ± 1.83	−1.17 ± 1.12	−1.44 ± 1.45	0.157
People’s reaction	−0.89 ± 1.34	−0.62 ± 1.03	−0.73 ± 1.03	0.462
Feeling about future	−3.48 ± 2.40	−3.60 ± 2.25	−3.02 ± 2.34	0.485
Financial situation	−0.85 ± 1.36	−0.74 ± 1.21	−0.61 ± 1.55	0.688
Living conditions	−0.66 ± 1.03	−0.50 ± 0.94	−0.56 ± 1.53	0.763
Dependence on others	−1.48 ± 1.87	−1.26 ± 1.36	−1.77 ± 1.64	0.374
Freedom to eat	−4.46 ± 2.68	−4.89 ± 2.67	−5.05 ± 2.97	0.518
Freedom to drink	−4.32 ± 2.98	−4.55 ± 2.90	−4.98 ± 3.20	0.556
AWI	−2.05 ± 1.08	−2.10 ± 0.90	−2.23 ± 1.27	0.716

Value is displayed as mean ± standard deviation. ADDQoL, Audit of Diabetes-Dependent Quality of Life; OAD, oral antidiabetic drug; QoL, quality of life; AWI, Average weighted impact score; ^*^p < 0.05.

A significant difference in the C-SOADAS and total scores was observed among the three groups (21.42 [1.98] vs. 20.43 [2.09] vs. 19.00 [2.24], *p* < 0.0001) (Tables 2, 4). The mean scores for the ability to control blood sugar, tolerability of the side effects, convenience of drug taking, and overall satisfaction were significantly higher in the group using two OADs than in the other two groups (*p* < 0.05). The effect of OADs on body weight was higher in the group with two OADs than in the groups with three and more than three OADs, with no statistically significant difference (3.98 [0.67] vs. 3.91 [0.50] vs. 3.73 [0.67], *p* = 0.1276).

**Table 4:** C-SOADAS in different oral antidiabetic drug groups.

Item	2 OADs (*n* = 65)	3 OADs (*n* = 47)	>3 OADs (*n* = 41)	*p* value
Q1: Ability to control blood sugar	4.44 ± 0.56	4.09 ± 0.75	3.93 ± 0.93	0.001
Q2: Effect of weight	3.98 ± 0.67	3.91 ± 0.50	3.73 ± 0.67	0.127
Q3: Tolerability of the side effect	4.43 ± 0.50	4.25 ± 0.49	4.10 ± 0.58	0.006
Q4: Convenience of drug taking	4.11 ± 0.59	3.96 ± 0.66	3.34 ± 0.85	<0.0001
Q5: Overall satisfaction	4.45 ± 0.53	4.21 ± 0.46	3.90 ± 0.62	<0.0001
Total C-SOADAS score	21.42 ± 1.98	20.43 ± 2.09	19.00 ± 2.24	<0.0001

Value is displayed as mean ± standard deviation. C-SOADAS, Chinese version of the Satisfaction with Oral Antidiabetic drugs; OADs, oral antidiabetic drugs; Q, question.

Regarding the correlation between the ADDQoL and AWI scores, as represented by Spearman’s rank correlation coefficient (*R_s_*), a weak correlation was observed between the general overall QOL and AWI scores. However, a statistically significant moderate correlation was observed between the impact of diabetes on specific aspects of life and AWI scores (*R_s_* = 0.350, *p* < 0.0001). The four factors of ADDQoL were moderately to highly correlated with the AWI scores (*p* < 0.0001), as shown in Supplementary Appendix 1. The ADDQoL and C-SOADAS scores showed correlations between QOL and treatment satisfaction (Supplementary Appendix 2). Among the two overview items of ADDQoL, the impact of overall QOL was positively correlated with the total C-SOADAS score, while the impact of diabetes on specific aspects of life was negatively correlated with the total C-SOADAS score, but neither reached a statistically significant difference. A significant low-to-moderate correlation was observed between the items of effect on weight, but no significant correlations were observed between the other items of treatment satisfaction.

## Discussion

The study results showed that patients with T2DM receiving metformin-based therapy using more than three OADs had the lowest HRQoL compared with those with less than or equal to three OADs. Furthermore, the treatment satisfaction of patients receiving two OADs was significantly higher than that of the other treatment combinations.

In the study, HbA1c was significantly higher in the group with more than three OADs (7.8% ± 0.9%) than in the other two groups, and 80% of those had poorer glycemic control (HbA1c ≥ 7%). These results are consistent with those of previous studies (15). A greater number of prescribed OADs together were correlated with more comorbidities, obesity, smoking, and high blood pressure, which increased insulin resistance. Previous studies showed that the use of multiple (>3) OADs for treating patients has been highly correlated with poor glycemic control and increasing duration of diabetes (16, 17). Furthermore, physical impairment has been observed in frail hypertensive older adults with hyperglycemia (18). Although this study included patients using OADs that had a negative impact on HRQoL, patients treated with two OADs had a higher HRQoL in relation to diabetes than those treated with three or more OADs.

Furthermore, this study revealed that whether patients used two, three, or more than three OADs, the domain of “dietary freedom” had the most significant effect on diabetes. A strong association was observed between diabetes and diet, and dietary control was involved in the management of T2DM as a major life factor and influenced the long-term outcomes of the disease, including depression (19, 20). Diabetes has a significant negative impact on diet, and the same results are seen in patients treated with OADs or insulin. Several studies on factors associated with diabetes treatment satisfaction showed that the use of insulin is negatively associated with treatment satisfaction (21). However, whether T2DM treated with OADs is positively or negatively associated with treatment satisfaction remains controversial and is only compared with dietary control (6, 22). These inconsistent results may be partly due to differences in the OADs tested.

However, the longer the duration of DM, the greater the number of OADs required for combination therapy owing to insufficient insulin secretion and poor glycemic control (15). Patients using OADs have been shown to have higher satisfaction levels than those using insulin. Furthermore, those treated with fewer drugs have significantly better satisfaction scores than those treated with multiple combination OADs (23, 24). The European multinational PANORAMA study (25) showed that the use of one or two and three or more OADs had no significant effect on treatment satisfaction compared with diet or exercise control, whereas the combination of OADs and insulin had significantly lower treatment satisfaction. However, a very limited number of studies have evaluated the treatment satisfaction of patients using various OAD classes. The study findings showed that the number of OAD classes was significantly associated with treatment satisfaction, and the treatment satisfaction scores decreased as the number of OADs increased. These results are consistent with those of previous studies showing that the use of more drugs may increase the complexity of the dosing regimen and may lead to other adverse drug reactions, such as weight gain and increased incidence of severe hypoglycemia, to achieve intensive glycemic control. Thus, poor adherence leads to worse treatment satisfaction (26). An analysis of the UK study on drug attribute preference in T2DM showed that the most important factors determining patients’ preference for OAD were the likelihood of hypoglycemic events, weight change, the likelihood of gastrointestinal side effects or nausea, especially for patients taking two or more drugs, and drug efficacy (27).

The AWI score reflects the overall impact of diabetes on a person’s life. The study results indicated that the mean weighted impact score had a low correlation with the overall (current) QOL score (*R_s_* = 0.148, *p* = 0.0682) and, as expected, a moderate correlation with the diabetes impact score on specific aspects of life (*R_s_* = 0.350, *p* < 0.0001). These results are similar to the results of the ADDQoL-CnTW validation study (12). The correlation between the AWI score and the diabetes-specific impact score (*R_s_* = 0.52, *p* < 0.01) was better than the overall QOL score (*R_s_* = 0.07, *p* > 0.05), which is consistent with the findings of previous similar studies, which showed that the diabetes-specific QOL psychometric instrument is more sensitive to individual changes. The European PANORAMA study (25), a cross-sectional investigation of 5,817 individuals, showed a higher correlation between AWI scores and diabetes impact scores on specific life aspects than overall QOL scores (*R_s_* = 0.21, *p* < 0.001 and *R_s_* = 0.60, *p* < 0.001). The 2016 Hong Kong study (11) suggests that the AWI score of the ADDQoL is less relevant to general generic instruments such as the SF-36 or EQ-5D for assessing a broad range of health states. A moderate-to-high correlation was observed between the mean weighted impact scores of the ADDQoL and the four factors of its instrument (*R_s_* = 0.490–0.916, *p* < 0.0001), which is consistent with the results of the ADDQoL-CnTW validation study (*R_s_* = 0.390–0.82) (12). Furthermore, a significant moderate-to-high correlation was observed between the mean weighted impact scores of the ADDQoL and 19 specific life domains in a Polish study (*R_s_* = 0.42–0.80, *p* < 0.001) (28). Therefore, it can be concluded that the ADDQoL can be used as a standard tool to measure diabetes-related QOL across ethnic groups, especially in relation to the impact of specific life domains of diabetes. Strengthening the appreciation of risk factors associated with HRQoL or treatment satisfaction has become an important program in diabetes healthcare. Assessing the association between treatment satisfaction and HRQoL may help healthcare providers identify patients’ perceptions of their disease, predict various aspects of the life of individuals with diabetes, and identify diabetes management that needs to be reinforced to improve treatment outcomes. However, few studies have evaluated the relationship between HRQoL and treatment satisfaction. To the best of our knowledge, this is the first study to evaluate the correlation between ADDQoL and C-SOADAS in patients with T2DM. This study showed a low positive correlation between the two, which is consistent with earlier findings. Although the questionnaires used in this study were inconsistent with previous studies in that they did not yield a significant correlation between HRQoL and treatment satisfaction, both the most commonly used general QOL measure (29) and the Diabetes-Related QOL Questionnaire (30) provided the same confirmation that perceptions of treatment satisfaction and perceptions and descriptions of burdens or limitations in QOL disagree, which suggests that treatment satisfaction and HRQoL are two distinct phenomena. Therefore, QOL and treatment satisfaction should be assessed concurrently in the comprehensive care of patients with diabetes.

### Strengths and limitations

To the best of our knowledge, this is the first study to investigate the QOL and treatment satisfaction of patients with T2DM receiving OADs in Taiwan. The first advantage of this study is that the majority of outpatients with diabetes in Taiwan use only OADs for blood glucose control, and this is increasing significantly. However, few outcome measures have been reported for diabetes-related patients using OADs alone. Second, this study used a validated, standardized instrument to assess diabetes-related QOL and treatment satisfaction. However, this study has several limitations.

First, the total sample size was relatively small. Patients with T2DM were recruited from a single medical center in southern Taiwan. Although the characteristics of the study population were similar to those of the Taiwan Annals of Diabetes data, it was not possible to address the issue of treatment patterns affecting the results and limit the generalizability of the study. Second, this cross-sectional study was inconclusive in establishing a causal relationship between sociodemographic and clinical characteristics, HRQoL, and treatment satisfaction, and assessing differences in changes in QOL at different time points was not feasible. However, previous studies have suggested that SGLT2 inhibitors can be anti-frailty drugs (31). Although 43% of our study population used SGLT2 inhibitors, we are uncertain about the effect of hypoglycemic drugs on QOL. Third, while respondents were encouraged to answer honestly to ensure that no relevant findings were affected, it may not be possible to completely avoid response bias in social expectations, especially for treatment satisfaction surveys, which is a common limitation of survey research. Fourth, previous studies showed a positive relationship between treatment satisfaction and HRQoL and medication adherence, especially in chronic diseases such as diabetes and hypertension (32). In this study, medication adherence was not explored, so bias in producing good glycemic control could not be avoided.

## Conclusion

This study evaluated the HRQoL and treatment satisfaction of patients with T2DM undergoing metformin-based treatment in combination with other OADs using the ADDQoL and C-SOADAS questionnaire in Taiwan. The results showed a significantly greater positive effect of fewer OAD classes on treatment satisfaction. Further studies with a larger sample size are needed to provide clinical healthcare providers with a more comprehensive understanding of the QOL and treatment satisfaction of patients with T2DM in Taiwan and worldwide.

## Data availability statement

The data analyzed in this study are subject to the following licenses/restrictions: First author had full access to all the data in the study and took responsibility for the integrity of the data and the accuracy of the data analysis. Data will be available with appropriate request to first author ((wun0910@gmail.com) or corresponding author by e-mail (jk2975525@hotmail.com). Requests to access these datasets should be directed to Y-WC, wun0910@gmail.com, and C-YC, jk2975525@hotmail.com.

## Ethics statement

This study was approved by the Institutional Review Board of the Chang Gung Medical Foundation (no. 202000024B0C501). All participants provided written informed consent. The patients/participants provided their written informed consent to participate in this study.

## Author contributions

Y-WC, F-CS, and C-YC were involved in the conception of the study, analysis, provided data, clinical information, and study design. All authors were involved in the interpretation of the findings, drafting the manuscript, reviewed, and approved the final manuscript and agreed to be held accountable for all aspects of the work.

## Funding

This study was funded by Kaohsiung Chang Gung Memorial Hospital (CMRP G8K0781).

## Conflict of interest

The authors declare that the research was conducted in the absence of any commercial or financial relationships that could be construed as a potential conflict of interest.

## Publisher’s note

All claims expressed in this article are solely those of the authors and do not necessarily represent those of their affiliated organizations, or those of the publisher, the editors and the reviewers. Any product that may be evaluated in this article, or claim that may be made by its manufacturer, is not guaranteed or endorsed by the publisher.

## Supplementary material

The Supplementary material for this article can be found online at: https://www.frontiersin.org/articles/10.3389/fpubh.2023.1152284/full#supplementary-material
