# Supplementary material for: Investigation of treatment satisfaction and health-related quality of life after add-on to metformin-based therapy in patients with type 2 diabetes
Source: Front Public Health. 2023 Apr 11;11:1152284. doi: 10.3389/fpubh.2023.1152284 (PMC10126269; doi:10.3389/fpubh.2023.1152284)
Supplement: Supplementary file 1 [file Table_1.docx]

**Supplementary Material**

Appendix 1. Relationship between ADDQoL and average weighted impact score

| Audit of Diabetes-Dependent Quality of Life | AWI (r_s_) |
| --- | --- |
| Overview questions |  |
| - 1. Present QoL score | 0.148 |
| 1. Diabetes-dependent QoL score | 0.350^*^ |
| 19 domain-specific items |  |
| F1: physical, social and leisure activities | 0.916^*^ |
| F2: attitudes about society reaction and life reaction and life circumstances | 0.737^*^ |
| F3: intimate relationships | 0.490^*^ |
| F4: diet | 0.659^*^ |
| ADDQoL, Audit of Diabetes-Dependent Quality of Life; AWI, average weighted impact; r_s_, Spearman's rank correlation coefficient; F, factor; ^*^: P<0.0001 | |
