# Supplementary material for: Investigation of treatment satisfaction and health-related quality of life after add-on to metformin-based therapy in patients with type 2 diabetes
Source: Front Public Health. 2023 Apr 11;11:1152284. doi: 10.3389/fpubh.2023.1152284 (PMC10126269; doi:10.3389/fpubh.2023.1152284)
Supplement: Supplementary file 2 [file Table_2.docx]

Appendix 2. Relationship between ADDQoL and C-SOADAS

|  |  | C-SOADAS (r_s_) | | | | | |
| --- | --- | --- | --- | --- | --- | --- | --- |
| ADDQoL | | Q1 | Q2 | Q3 | Q4 | Q5 | Total |
|  |  | Ability to control blood sugar | Effect on weight | Tolerability  of the side effects | Convenience of drug taking | Overall satisfaction |  |
| **Overview questions** | |  |  |  |  |  |  |
| Present QoL score | | 0.125 | 0.045 | 0.135 | 0.023 | 0.104 | 0.109 |
| Diabetes-dependent QoL score | | -0.004 | -0.002 | -0.024 | -0.046 | -0.043 | -0.022 |
| **19 domain-specific items** | |  |  |  |  |  |  |
| **F1: physical, social and leisure activities** | | 0.073 | 0.225^*^ | 0.031 | 0.076 | 0.057 | 0.121 |
| Leisure activities | | 0.174^*^ | 0.175^*^ | 0.020 | 0.066 | 0.231^*^ | 0.182^*^ |
| Working life | | 0.140 | 0.251^*^ | 0.071 | 0.081 | 0.221^*^ | 0.211 |
| Journeys | | 0.128 | 0.209^*^ | -0.002 | 0.049 | 0.095 | 0.126 |
| Holidays | | 0.056 | 0.224^*^ | -0.018 | 0.090 | 0.073 | 0.117 |
| Physical health | | -0.023 | 0.159 | 0.017 | -0.015 | -0.061 | 0.012 |
| Family life | | -0.054 | -0.004 | 0.003 | 0.062 | 0.053 | 0.030 |
| Friendship and social life | | 0.023 | 0.181 | 0.068 | 0.095 | -0.053 | 0.072 |
| Physical appearance | | 0.095 | 0.154 | 0.128 | 0.016 | 0.136 | 0.121 |
| Motivation | | 0.018 | 0.125 | -0.046 | -0.007 | -0.069 | -0.022 |
| Feeling about future | | 0.045 | 0.130 | -0.002 | 0.032 | -0.039 | 0.054 |
| **F2: attitudes about society reaction and life reaction and life circumstances** | | 0.104 | 0.161^*^ | 0.052 | 0.073 | 0.025 | 0.102 |
| Self-confidence | | 0.005 | 0.105 | -0.016 | 0.011 | -0.002 | 0.029 |
| People’s reaction | | 0.027 | 0.103 | 0.045 | 0.066 | 0.009 | 0.051 |
| Financial situation | | 0.112 | 0.141 | 0.108 | 0.049 | 0.019 | 0.136 |
| Living conditions | | 0.099 | 0.178^*^ | 0.101 | 0.054 | -0.057 | 0.091 |
| Dependence on others | | 0.054 | 0.067 | 0.047 | 0.024 | 0.027 | 0.048 |
| **F3: intimate relationships** | | 0.146 | 0.232^*^ | 0.105 | 0.092 | 0.167 | 0.230^*^ |
| Close personal relationship | | 0.183^*^ | 0.207^*^ | 0.118 | 0.144 | 0.235^*^ | 0.274^*^ |
| Sex life | | 0.182 | 0.225^*^ | 0.108 | 0.157 | 0.105 | 0.225^*^ |
| **F4: diet** | | 0.124 | 0.120 | 0.163^*^ | 0.106 | 0.071 | 0.130 |
| Freedom to eat | | 0.153 | 0.135 | 0.160^*^ | 0.091 | 0.026 | 0.120 |
| Freedom to drink | | 0.075 | 0.083 | 0.144 | 0.099 | 0.108 | 0.120 |
| **AWI score** | | 0.152 | 0.247^*^ | 0.093 | 0.125 | 0.088 | 0.189^*^ |
| ADDQoL, Audit of Diabetes-Dependent Quality of Life; C-SOADAS, Chinese version of Satisfaction with Oral Anti-diabetic Agent Scale; AWI, average weight impact ; r_s_, Spearman's rank correlation coefficient; *, P<0.05. | | | | | | | |
